# Variability in Swallowing Biomechanics in Infants with Feeding Difficulties: A Videofluoroscopic Analysis

**DOI:** 10.1007/s00455-022-10436-2

**Published:** 2022-03-17

**Authors:** Laura Fuller, Anna Miles, Isuru Dharmarathna, Jacqui Allen

**Affiliations:** 1 Speech Science, School of Psychology, The University of Auckland, Grafton Campus, Private Bag 92019, Auckland, New Zealand; 2 Counties Manukau District Health Board, Auckland, New Zealand

**Keywords:** Paediatric feeding disorder, Aspiration, Videofluoroscopic swallow study, Variability, Infants

## Abstract

Clinicians performing feeding evaluations in infants often report swallow variability or inconsistency as concerning. However, little is known about whether this represents pathological incoordination or normal physiologic variance in a developing child. Our retrospective study explored quantitative videofluoroscopic measures in 50 bottle-fed infants (0–9 months) referred with feeding concerns. Our research questions were as follows: Is it possible to assess swallow to swallow variability in an infant with feeding concerns, is there variability in pharyngeal timing and displacement in infants referred for videofluoroscopy, and is variability associated with aspiration risk? Measures were taken from a mid-feed, 20-s loop recorded at 30 frames per second. Each swallow within the 20-s loop (*n* = 349 swallows) was analysed using quantitative digital measures of timing, displacement and coordination (Swallowtail™). Two blinded raters measured all swallows with strong inter-rater reliability (ICC .78). Swallow frequency, suck-swallow ratio, residue and aspiration were also rated. Variability in timing and displacement was identified across all infants but did not correlate with aspiration (*p* > .05). Sixteen infants (32%) aspirated. Across the cohort, swallow frequency varied from 1 to 15 within the 20-s loops; suck-swallow ratios varied from 1:1 to 6:1. Within-infant variability in suck-swallow ratios was associated with higher penetration-aspiration scores (*p* < .001). In conclusion, pharyngeal timing and displacement variability is present in infants referred with feeding difficulties but does not correlate with aspiration. Suck-swallow ratio variability, however, is an important risk factor for aspiration that can be observed at bedside without radiation. These objective measures provide insight into infant swallowing biomechanics and deserve further exploration for their clinical applicability.

## Introduction

Swallowing is a complex process that requires the precise coordination of 26 groups of muscles and the integration of the respiratory, gastrointestinal, masticatory and neurological systems [1–3]. Liquid feeds in young infants show significant physiological differences to eating and drinking in older children and adults. The reflexive, motor pattern of suckling in an infant is a result of the coordination of the lips, tongue, jaw, palate, hyoid bone, and pharynx and, it is understood that the suckling reflex diminishes around 4–6 months of age as textures are introduced and swallowing comes under more volitional control [1–3]. Whilst infant feeding is considered primarily reflexive, the process of swallowing has inherent variability with changes in hunger / thirst, state, viscosity, flow rate, volumes, general fatigue, as well as transience in reflexes and the masticatory demand over a mealtime as a child introduces textures [4, 5]. The presence of adaptability in suckling and sucking patterns is supported by work in breastfeeding which found wide ranges in measures within and between breastfeeds [6–11]. This suggests that cortical modulation is possible and one interpretation is that this variability in swallows during breastfeeding is an adaptation to the variable flow from the breast [6–11]. With this inherent adaptability, swallow to swallow differences are to be expected. Variability or cortical modulation is considered a common feature of healthy swallowing in adults and a sign of a flexible system that responds to bolus properties [12–14]. Yet, in paediatric feeding, clinicians often report swallow variability or inconsistency in infants as a sign of concern in feeding evaluations, describing the swallow as ‘incoordinated’. In infants, where milk suckling is a more reflexive, rhythmical process, can variability be excessive and therefore pathological with an impact on safety? Or, similar to adults, is a lack of variability a sign of inability to adapt and modulate and so a sign of safety risk? Given that these two scenario’s represent opposite interpretations, a greater understanding of feeding biomechanics and in particular variability in infants would greatly improve the accuracy of assessment and guide tailored interventions.

Videofluoroscopic swallowing study (VFSS) is one of the most common instrumental feeding assessments across the lifespan because it allows for the visualization of all phases of swallowing. By using objective measures, we are able to capture patterns of movement in a reliable, repeatable manner. Our lab has previously validated these measures in infants and children [15–19]. These measures allow between infant, and within-infant variability to be measured quantitatively. This study explored variability in swallowing biomechanics in infants with feeding difficulties using videofluoroscopic analysis. Our research questions were as follows: Is it possible to assess swallow to swallow variability in an infant with feeding concerns? Is there variability in pharyngeal timing and displacement in infants referred for videofluoroscopy, and is variability associated with aspiration risk?

## Methods

This study received appropriate regional ethics and locality approval by The University of Auckland Human Patients Ethics Committee New Zealand (9263). Infants consecutively referred to the radiology department at Starship Childrens’ Health or Middlemore Hospital with concerns regarding feeding were included. Inclusion criteria were under 9 months old, full 20-s mid-feed loop, ability to view oropharyngeal anatomy and physiology. Studies that needed to be terminated prior to 20 s due to aspiration or non-compliance were excluded. Information regarding medical history, demographics, presence of a tracheostomy, history of lower respiratory tract infections (LRTI) and oxygen requirement were collected. Infants were broadly categorised by their corrected chronological age (group 1: 0–2 months, group 2: 3–5 months, group 3: 6–9 months) and grouped by their primary medical conditions into: neurological (including hypoxic ischaemic encephalopathy and neonatal drug exposure), respiratory (chronic lung disease and bronchiolitis), cardiac (valve surgery), anatomical (cleft palate), chromosomal (Down Syndrome) and unknown (typically developing with feeding concerns with no known medical aetiology) groups.

VFSS was conducted in the radiology suite on a Siemens Sireskop radiographic unit (Siemens, Munich, Germany). Either breast milk or recommended formula was used to prepare materials for the study, according to the particular infant’s needs. We used Varibar barium sulphate contrast (40% w/v) (E-Z-EM Canada Inc, Quebec, Canada) in 50:50 to create Level 0 Thin fluids (International Dysphagia Diet Standardization Initiative, IDDSI, 2016). Infants were placed at their usual or recommended feeding posture with the support of a caregiver/ parent and used their usual nipple and bottle. 20-s video loops of ‘midfeed’ bottle feeding were recorded. ‘Midfeed’ was defined as midway through the feed, ensuring that infants had established their stable, functional feeding pattern. The timing of recording the midfeed loop was determined by the attending speech pathologist and was recorded on a USB external drive in.avi file format at 30 frames per second (f/s) for frame-by-frame analysis.

**Fig. 1:**
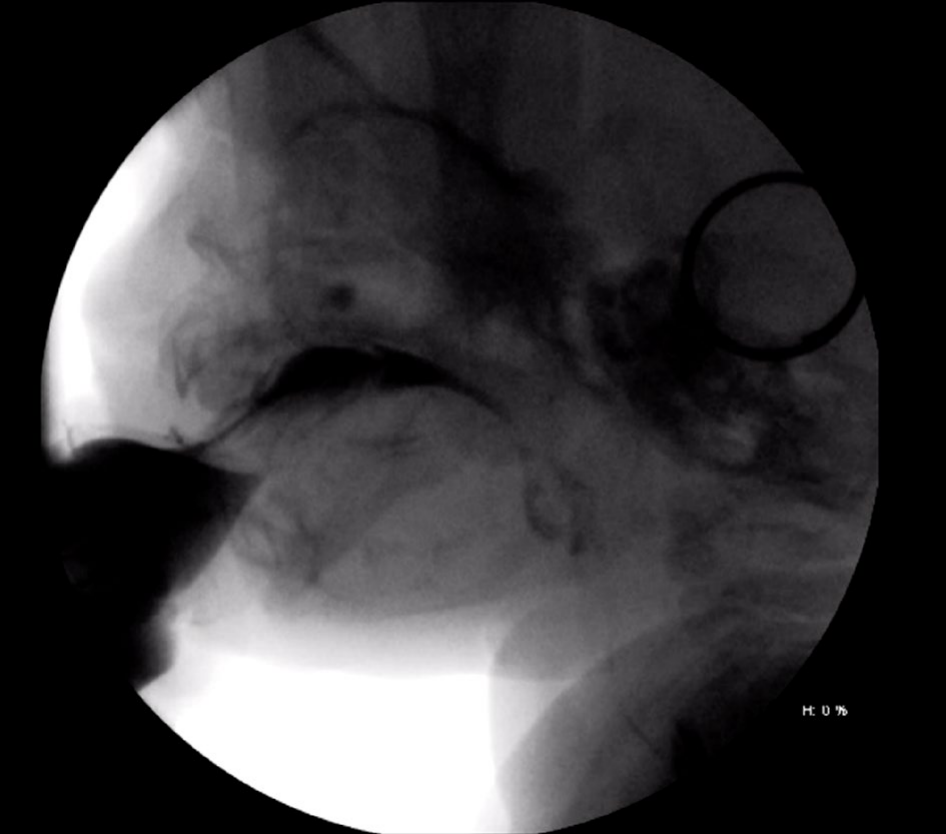
Example of a VFSS image of an infant where airway view is obscured by positioning

The videos were analysed using Swallowtail™ (Belldev Medical, Chicago, USA). Each swallow was analysed utilizing the timing, line and area features in the program. The embedded timing and displacement measures were developed by Leonard and Kendall [20] and adapted by members of our lab to reflect the paediatric population including the addition of number of swallows and suck to swallow ratios. All measures have proven reliability and validity [15–18]. Other key clinical notations were measured in binary form including the presence of residue, nasopharyngeal regurgitation and presence of gastroesophageal reflux. The Penetration-Aspiration Scale [21] was used to reflect current clinical practice and to draw on functional relevance in identifying airway safety during swallowing. Each swallow was given a Penetration-Aspiration Scale score. Airway risk was also rated for each swallow following the rating system of scores > 3 on the Penetration-Aspiration Scale described by Steele and colleagues [22]. A binary measure of aspiration was calculated for each individual (PAS < 3 versus PAS 3 +). All swallows were measured twice by two blinded independent raters. Raters were experienced speech pathologists in infant VFSS and had received training, and had measured > 100 videos using objective quantitative parameters. Hyoid measures were not taken as the hyoid bone has been proven to be difficult to visualize in those < 9 months old [19]. Where the airway was difficult to view, or the infant did not return to resting position with open airway between swallows, airway measures were not taken (Fig. 1).

### Data Analysis

Using Excel (Microsoft, Seattle, USA), descriptive statistics were calculated. Data were then transferred to IBM SPSS Statistics V24 (SPSS inc. Chicago, Illinois, USA) for further analysis. Histograms and normality testing demonstrated that the data were not normally distributed, and standard error (SE) was selected as a measure of variability for statistical analysis. Non-parametric correlation statistics (Spearman, Chi-Squared and Point Biserial depending on data type) were used to assess relationships between infants’ SE scores across variables (objective measures, dysphagia symptoms and demographics). Non-parametric differences in SE were explored across diagnostic groups and aspirating versus non-aspirating infants using Mann Whitney U Test. Relationships with < 0.05 probability level were considered significant. Cohen's kappa test was used to assess inter-rater reliability for categorical measures, and intra-class coefficient (ICC) with 95% confidence intervals (CIs) used for continuous measures.

## Results

Infants swallowed between 1 and 15 times during the 20-s mid-feed loop with a total of 349 swallows for analysis from the 50 selected infants. There were three outliers who only swallowed once within the 20-s loop. These infants were excluded from further analyses in view of the study aim to investigate swallow variability within an infant and to reduce data skewness. The characteristics of the 50 infants are displayed in Table 1.

**Table 1:** Infant demographics (*n* = 50)

Variable	*n*	%
*Gender*		
Female	14	28
Male	36	72
*Age*		
0–3 months	16	32
4–6 months	13	26
7–9 months	21	42
*Primary medical diagnosis*		
Neurological including epilepsy, meningioma	9	18
Respiratory including bronchomalacia, chronic lung prematurity, bronchiolitis	17	34
Cardiac surgery	2	4
Anatomical including cleft palate, laryngomalacia	5	10
Chromosomal including Down’s syndrome	4	8
Unknown including low birth weight, nil diagnosis	13	26
*Oxygen support required via nasal prongs (low-flow only)*		
Yes	9	18
No	41	82
*Nasogastric tube*		
Present	20	40
Not present	30	60
*Tracheostomy*		
Present	1	2
Not present	49	98
*Presence of current respiratory illness*		
Yes	21	42
No	29	58
*Airway compromise*		
Present (PAS = 3–8)	16	32
PAS 5–8 (aspirators)	11	22
Not present (PAS = 1 or 2)	34	68

The objective measures gained from the cohort are displayed in Table 2, comparing values across all infants (excluding 3 outliers who swallowed only once in the 20-s loop). Inter-rater reliability was substantial across all measures. All binary subjective measures and the Penetration-Aspiration Scale achieved 100% agreement using Cohen's κ test. A moderate-strong degree of reliability was found between raters on all timing measures (ICC = 0.78, 95% CI 0.73-0.82 *p* < 0.001). A high degree of reliability was also found between raters on all displacement measures (ICC = 0.82, 95% CI 0.78-0.84, *p* < 0.001).

**Table 2:** Population variability: swallowing measures for all swallows across all 47 infants (*n* = 346 swallows)* excluding the 3 outliners

Quantitative measures *(based on *[20]*)*		Min	Max	*Mean*	*SE*
Timing measures	Total pharyngeal transit time (TPT)	0.3	0.57	0.49	0.1
	Time to airway closure* (AEs-AEcl)	0.03	1.1	0.62	0.4
	PES opening duration (PESdur)	0.23	0.31	0.24	0.1
	SP closure duration (T-SP)	0	3.82	1.6	0.51
Coordination measures	Bolus head reaches PES in relation to frame of complete airway closure (B1-AEcl)*	0.07	0.4	0.1	0.1
Displacement measures	Pharyngeal constriction ratio (PCR)	0	0.46	0.1	0
	Pharyngoesophageal segment maximum opening (PESmax)	0.2	0.6	0.4	0.1

Descriptive swallow measures		
Penetration-aspiration scale score	Median 1; Mode: 1, range 1–8	
Frequency of Pen/Asp (PAS 3 +)	65/349	32% of infants 18% of swallows
Residue present	42/349	12% of swallows
Naso-pharyngeal regurgitation (NPR) present	24/349	7% of swallows
Pharyngo-esophageal regurgitation (PER) present	22/349	6% of swallows

Suck swallow measures	Median	Max	Mode
Number of swallows in 20 s	7	15	10
Number of sucks in 20 s	10	17	10
Suck: swallow ratio	2:1	6:1	1:1

*Missing 84 data points where measure could not be taken

**Fig. 2:**
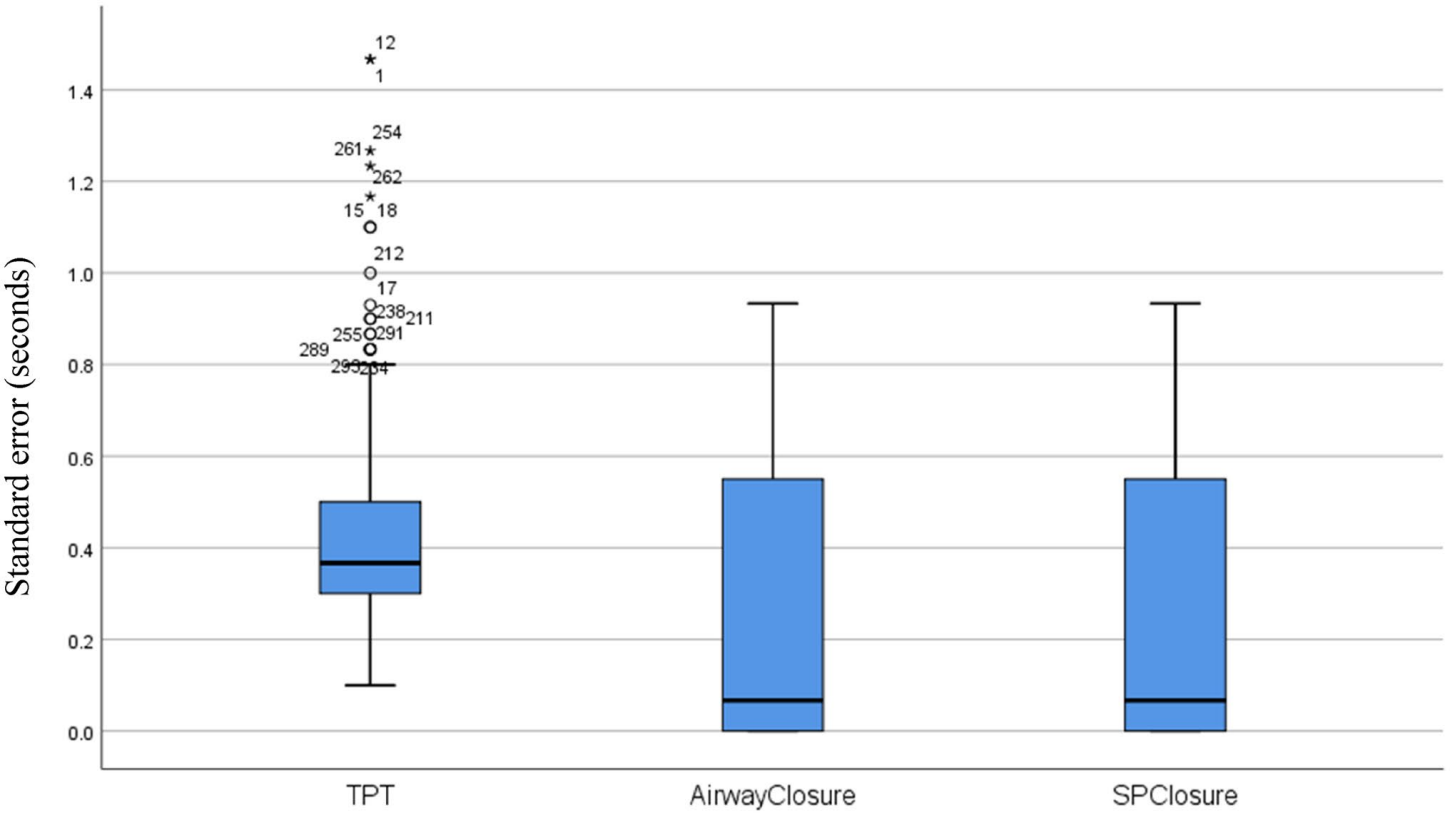
Timing measures demonstrating the variability of all scores (SE). *TPT* total pharyngeal transit time, *AirwayClosure* time to airway closure (AEs-AEcl), *SPclosure* tongue and soft palate cycle (T-SP)

**Fig. 3:**
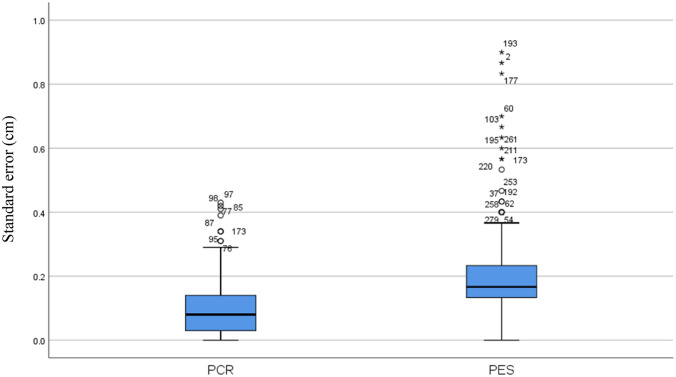
Displacement measures demonstrating the variability of all scores (SE). *PCR* pharyngeal constriction ratio, *PES* pharyngoesophageal segment maximum opening

There was significant within-infant variability in both timing and displacement measures (Figs. 2, 3). Within swallow timing and displacement measures correlated with one another (Table 3). There was no significant correlation between age and variability in objective swallowing measures (*p* > 0.05). There were no statistically significant differences in timing and displacement measures between diagnostic groups (*U* = 0.001, *p* = 0.317). Suck to swallow ratio was higher in those with neurology as their primary medical diagnosis (Median 4:1 suck to swallow ratio) when compared to those with a respiratory diagnosis (Median 2:1 suck to swallow ratio) (*U* = 36.00, *p* < 0.05).

**Table 3:** Correlations between measures

	TPT	AEs-AEcl	SP	PESdur	PCR	Number swallows	Pen/Asp	Sucks:swallow
TPT		***r***_**s**_** = .30, *p* < .05**	*r*_s_ = .20, *p* > .05	***r***_**s**_** = .54, *p* < .05**	***r***_**s**_** = .40, *p* < *.*05**	*r*_s_ = .07, *p* > .05	*r*_s_ = .19, *p* > .05	*r*_s_ = .11, *p* > .05
AEs-AEcl	***r***_**s**_** = .30, *p* < *.*05**		***r***_**s**_** = .68, *p* < *.*05**	***r***_**s**_** = .29, *p* < *.*05**	***r***_**s**_** = .76 *p* < *.*05**	*r*_s_ = .30, *p* > .05	*r*_s_ = -.30, *p* > .05	*r*_s_ = -.07, *p* > .05
T-SP	*r*_s_ = .20, *p* > .05	***r***_**s**_** = .68, *p* < *.*05**		*r*_s_ = .10, *p* > *.*05	***r***_**s**_** = .69, *p* < *.*05**	*r*_s_ = -.06, *p* > .05	*r*_s_ = .18, *p* > .05	*r*_s_ = .23, *p* > .05
PES dur	***r***_**s**_** = .354 *p* < *.*05**	***r***_**s**_** = .29, *p* < *.*05**	*r*_s_ = .10, *p* > *.*05		*r*_s_ = .35, *p* > *.*05	*r*_s_ = .01, *p* > .05	*r*_s_ = .07, *p* > .05	*r*_s_ = -.01, *p* > .05
PCR	***r***_**s**_** = .40, *p* < *.*05**	***r***_**s**_** = .76, *p* < *.*05**	***r***_**s**_** = .69, *p* < *.*05**	*r*_s_ = .35, *p* > *.*05		*r*_s_ = -.08, *p* > 0.05	*r*_s_ = -.19, *p* > .05	*r*_s_ = .12, *p* > .05
Number swallows	*r*_s_ = .07, *p* > .05	*r*_s_ = .30, *p* > .05	*r*_s_ = .06, *p* > .05	*r*_s_ = .01, *p* > .05	*r*_s_ = .08, *p* > .05		*r*_s_ = -.13, *p* > .05	***r***_**s**_** = -.61, *p* < .05**
Pen-Aspiration Scale	*r*_s_ = .26, *p* > .05	*r*_s_ = .11, *p* > .05	*r*_s_ = .16, *p* > .05	*r*_s_ = .18, *p* > .05	*r*_s_ = .26, *p* > .05	*r*_s_ = -13., *p* > .05		***r***_**s**_** = .28, *p* < .05**
Suck:swallow	*r*_s_ = .11, *p* > .05	*r*_s_ = .07, *p* > .05	*r*_s_ = 231, *p* > .05	*r*_s_ = .01, *p* > .05	*r*_s_ = .12, *p* > .05	***r***_**s**_** = -.61, *p* < .05**	***r***_**s**_** = .28, *p* < .05**	

Bold represents a statistical significance

*TPT* total pharyngeal transit time, *AEs-AEcl* time to airway closure, *T-SP* tongue and soft palate cycle, *PESdur* PES opening duration, *PCR* pharyngeal constriction ratio

### At-Risk Swallows

Aspiration occurred in 18% of all swallows measured, and 32% of all infants assessed. In infants who aspirated, no infant aspirated with every swallow. Increasing age showed a negative correlation with aspiration (*r*_s_ = − 0.28 *p* < *0.05*) with 38% of infants under 3 months aspirating, 38% of infants between 4 and 6 months of age aspirating and 18% of infants between 6 and 9 months of age aspirating. Airway violation, including binary scores (PAS < 3 versus ≥ 3) and penetration-aspiration scale raw scores did not correlate with timing and displacement variability measures (Table 3). There were no statistically significantly differences in timing and displacement variability measures between aspirating vs. non-aspirating infants (*U* = 0.021, *p* = *0.317*). There was a significant association between suck to swallow ratio and aspiration, with only 33% of infants’ swallows with < 3 sucks per swallow leading to aspiration compared with 66% of infants’ swallows with > 3 sucks per swallow. Suck to swallow ratio SE was also significantly associated with an infant’s maximum penetration-aspiration scale score suggesting that variance in suck to swallow ratio is associated with a greater aspiration risk (*r*_s_ = 0.28, *p* < 0.01).

## Discussion

This exploratory study examined variability in objective videofluoroscopic timing and displacement measures in 50 infants referred with feeding concerns. Objective measures and variability in measures were recorded successfully with moderate-strong inter-rater reliability. By choosing a mid-feed loop, variability associated with ‘warming up’ was considered to be reduced [23], leaving mid-feed biomechanics to be observed. Interestingly, whilst variability was seen in timing and displacement in this cohort, measures did not correlate with aspiration. Aspiration was, however, associated with a higher suck:swallow ratio, particularly if suck:swallow ratio was > 3 sucks per swallow, and with variability in suck:swallow ratio within a 20-s mid-feed loop. The population frequency of aspiration observed in this study (32%) is congruent with other patient cohort studies [15]. Aspiration was not a constant with some infants aspirating only once, and others aspirating across more swallows. No infant aspirated on all swallows. This has also been seen in adult populations with dysphagia [24].

### Variability in Measures

The quantitative data presented demonstrated variability, reliability and some parallels with adult data. Infants demonstrated variability in timing and displacement within this 20-s loop and absence of a correlation between variability and aspiration. This is congruent with research in healthy adults [12–14]. There are significant anatomical differences between adults and infants, and this was a cohort of infants with feeding concerns, so parallels must be drawn with caution. The exact degree of variability that is ‘typical’ is yet to be defined. This study presents preliminary findings on the presence of variability and feasibility of measures capturing this. It is possible that changes in the swallowing pattern could also be consistent with motor learning. Previous research has suggested that a new skill is learned through trial resulting in error and subsequent error modulation [25]. Therefore, variability may represent the infant ‘practicing’ and refining swallowing patterns and learning to correct errors of unsafe patterns. Other systems within the infant that contribute to swallow modulation are also developing at this age, including the respiratory system, peripheral nervous system and central nervous system [2, 26, 27]. Variability may also represent skill development and system maturation. Geddes et al. propose that a reduction of single sucks during feeding indicates developmental feeding progression [6], and supports the concept of swallow maturation and skilfulness. A relationship between variability and age was not seen in our small cohort of 50 infants and larger studies are needed to further explore this. It is also possible though, that the variability recorded may represent pathological inconsistency and clarification would require a large normative dataset for comparison.

### Suck-Swallow Ratio and Aspiration

Our previous published work in 146 infants found longer total pharyngeal transit time (TPT) and delayed airway closure in relation to bolus position at the PES (BP1AEcl) in infants who aspirated, suggesting that timing of swallows plays an important role in airway safety [18]. This current study demonstrated that whilst timing may be associated with aspiration in infants, *variability* in timing and displacement is not. Previous work also identified infants that took more than three sucks per swallow had an increased risk of aspiration [18]. Our current study builds on our understanding of how much *variability* in *suck:swallow ratio* may be associated with increased risk of aspiration. This is a useful finding as counting sucks per swallow is easily applicable to clinical practice and may increase the accuracy of identifying those at risk of airway compromise during bedside feeding evaluations.

### Limitation and Future directions

This study has limitations. Like many studies in paediatric feeding disorders, this study had a small sample size, and on this basis, findings must be interpreted with caution [23, 28]. We report corrected chronological age in participants and gestational age was not recorded. Rather than reporting prematurity, we report broad medical aetiology, however, many of these infants would have been born prematurely. Future studies would benefit from reporting prematurity and gestational age as well as a more extensive age range and sample size in order to establish higher levels of confidence.

Many factors will impact the swallowing patterns of young infants including intrinsic factors, environmental factors and imposed factors. Unlike in adult studies, where bolus volume can be easily measured and metered, this study did not measure volumes extracted from the bottle by infants and variability may be in response to volume changes. Methodologies that allow volumetric control or monitoring would be valuable. Exploring changes in variability at the beginning, middle and end of a feed may be interesting study avenue and identify whether fatigue plays a role in variability measures. Additional metrics that could be considered for correlation with videofluoroscopic findings in future research include manometry, tongue pressure, bolus volume and respiratory measures.

The quantitative methods used in this study allow specific biomechanical measures of timing and displacement ideal for this type of research exploration. We are not expecting clinicians to measure every swallow in their infants to look for variability. This was a research exploration of variability not an attempt at developing a clinical tool. We are exploring a conceptual idea to see if we can understand the range of behaviours involved in infant swallowing across the developing infant ages. Extrapolating information about normal variance must be taken with caution in this cohort of infants referred with feeding concerns. However, this offers some early evidence of variance in a climate where radiographic data on normal infants are not available nor deemed ethical to collect.

### Conclusion

The present study demonstrates the reliability of objective quantitative swallowing measures in examining intra-subject variability in swallowing in infants with feeding concerns. Whilst timing and displacement measures were variable between infants and within the same infant, there was no relationship between variance and aspiration. A 20-s mid-feed videofluoroscopic loop provided ample opportunity to observe aspiration frequency and suck-swallow ratio variability in most infants. Single swallows are insufficient to rule out aspiration, and longer sequences of swallows are more likely to reflect actual ability. Consistent with previous data from our laboratory, infants taking > 3 sucks per swallow demonstrated significantly increased risk of aspiration. In a mid-feed swallow, variability in suck:swallow ratios within a 20-s loop was also significantly associated with increased the risk of aspiration. Quantitative measures help our understanding of the patterns of variability in infants. Future use of objective swallowing measures may further clarify infant dysphagia diagnosis and provide a more accurate avenue for treatment planning.
